# Pore dilation occurs in TRPA1 but not in TRPM8 channels

**DOI:** 10.1186/1744-8069-5-3

**Published:** 2009-01-21

**Authors:** Jun Chen, Donghee Kim, Bruce R Bianchi, Eric J Cavanaugh, Connie R Faltynek, Philip R Kym, Regina M Reilly

**Affiliations:** 1 Neuroscience, Global Pharmaceutical Research and Development, Abbott Laboratories, Abbott Park, IL 60064-6125, USA; 2 Department of Physiology, Rosalind Franklin University of Medicine and Science, The Chicago Medical School, 3333 Green Bay Road, North Chicago, IL60064, USA

## Abstract

Abundantly expressed in pain-sensing neurons, TRPV1, TRPA1 and TRPM8 are major cellular sensors of thermal, chemical and mechanical stimuli. The function of these ion channels has been attributed to their selective permeation of small cations (e.g., Ca^2+^, Na^+ ^and K^+^), and the ion selectivity has been assumed to be an invariant fingerprint to a given channel. However, for TRPV1, the notion of invariant ion selectivity has been revised recently. When activated, TRPV1 undergoes time and agonist-dependent pore dilation, allowing permeation of large organic cations such as Yo-Pro and NMDG^+^. The pore dilation is of physiological importance, and has been exploited to specifically silence TRPV1-positive sensory neurons. It is unknown whether TRPA1 and TRPM8 undergo pore dilation. Here we show that TRPA1 activation by reactive or non-reactive agonists induces Yo-Pro uptake, which can be blocked by TRPA1 antagonists. In outside-out patch recordings using NMDG^+ ^as the sole external cation and Na^+ ^as the internal cation, TRPA1 activation results in dynamic changes in permeability to NMDG^+^. In contrast, TRPM8 activation does not produce either Yo-Pro uptake or significant change in ion selectivity. Hence, pore dilation occurs in TRPA1, but not in TRPM8 channels.

## Background

Abundantly expressed in sensory neurons, TRPV1, TRPA1 and TRPM8 are involved in sensory function, pain and neurogenic inflammation [1]. The function of these ion channels has been attributed to their ability to pass certain ion species across the plasma membrane. Once activated, TRPV1, TRPA1 and TRPM8 are permeable to small cations such as Ca^2+^, K^+^, Na^+^; hence, channel activation simultaneously depolarizes the plasma membrane and raises intracellular Ca^2+^, which subsequently triggers a variety of physiological processes. By analogy to voltage-gated K^+ ^channels, it is assumed that ion selectivity of TRP channels should be an invariant signature to the respective channel. However, this notion has been challenged recently. When activated, TRPV1 exhibits time and agonist-dependent changes in ion selectivity [2]. In fact, TRPV1 undergoes pore dilation and allows permeation of large organic cations, including spermine (202.3 Da), NMDG (195.2 Da), Yo-Pro (376 Da), gentamycin (477.6 Da) and QX-314 [3-7]. Here we explored whether TRPA1 and TRPM8 undergo pore dilation by examining Yo-Pro uptake and changes in ion selectivity upon channel activation.

## Results and discussion

**Figure 1:**
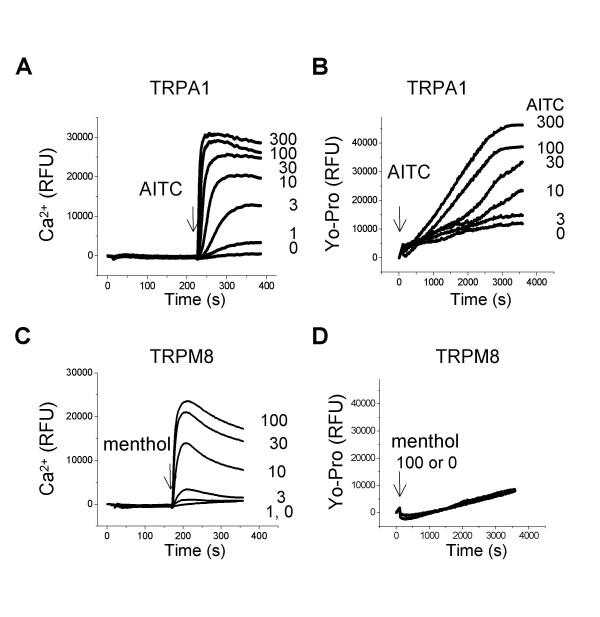
**The activation of TRPA1, but not TRPM8, induced Yo-Pro uptake**. A, in HEK-293F cells expressing rat TRPA1, AITC elevated intracellular Ca^2+^, as represented by increases of fluorescence signals (RFU) in the FLIPR based Ca^2+ ^assay. B, in cells expressing TRPA1, AITC evoked robust Yo-Pro uptake in a concentration-dependent manner from the FLIPR based Yo-Pro uptake assays. C, in cells expressing human TRPM8, menthol activated TRPM8 and elevated intracellular Ca^2+^. D, in cells expressing TRPM8, menthol failed to induce Yo-Pro uptake. Compounds are in μM and additions are indicated by arrows.

Yo-Pro is a divalent cation impermeable to the plasma membrane. However, under certain conditions, it can enter cells, bind nucleic acids and emit fluorescence. Hence the uptake of Yo-Pro has been used previously as an indicator of pore dilation [2,8,9]. In HEK293-F cells transiently expressing rat TRPA1, allyl isothiocyanate (AITC) evoked robust increases in intracellular Ca^2+ ^(Fig. 1A). Concomitantly, AITC also induced Yo-Pro uptake in a concentration-dependent manner (Fig. 1B). At higher concentrations of AITC (100 or 300 μM), the increase in fluorescence was immediately noticeable and continued to increase for about 50 min. In addition, AITC also induced Ca^2+ ^influx and Yo-Pro uptake in cells expressing human TRPA1 and mouse TRPA1, but not in untransfected cells (data not shown). In cells expressing human TRPM8, menthol activated TRPM8 as indicated by the concentration-dependent Ca^2+ ^influx, but failed to induce Yo-Pro uptake (Fig. 1C and 1D). Other TRPM8 agonists (e.g., icilin) also evoked Ca^2+ ^influx but failed to induce Yo-Pro uptake (data not shown). Hence, Yo-Pro uptake occurs upon activation of TRPA1, but not TRPM8.

**Figure 2:**
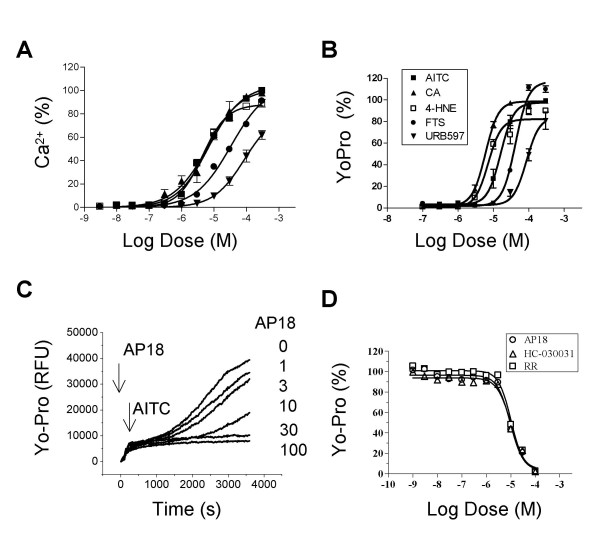
**Yo-Pro uptake was evoked by various TRPA1 agonists and blocked by TRPA1 antagonists**. Concentration-effect relationships for agonist responses in the Ca^2+ ^assay (A) and Yo-Pro uptake (B). Reactive agonists: AITC, CA and 4-HNE. Non-reactive agonists: FTS and URB597. Data are represented as percentage of maximal AITC responses. C, representative traces of Yo-Pro uptake in response to a first addition of AP18 (0 to 100 μM) and a second addition of AITC (30 μM). AP18 inhibited AITC evoked Yo-Pro uptake in a concentration-dependent manner. D, concentration-effect relationship of Yo-Pro uptake inhibition by AP18, HC-030031 and RR. (n = 4 – 8).

In addition to AITC, TRPA1 can be activated by many other electrophilic agonists (e.g., cinnamaldehyde or CA, 4-hydroxynonenal or 4-HNE), and non-reactive agonists (e.g., URB597, farnesyl thiosalicylic acid or FTS) [10-14]. We investigated whether the Yo-Pro uptake is limited to AITC. CA, 4-HNE, FTS and URB597 all evoked Ca^2+ ^influx and Yo-Pro uptake in a concentration dependent-manner (Fig. 2A and 2B). In the Ca^2+ ^assay, the EC_50 _was 6.5 ± 0.35 μM for AITC, 6.8 ± 1.5 μM for CA, 4.4 ± 0.6 μM for 4-HNE, 33.2 ± 8.1 μM for FTS and 85.6 ± 10.4 μM for URB597 (n = 4–8). Compared to AITC, the maximal signals were 104% for CA, 88% for 4-HNE, 107% for FTS and 82% for URB597. In the Yo-Pro uptake assay, the EC_50 _was 16.0 ± 3.8 μM for AITC, 5.9 ± 0.7 μM for CA, 7.1 ± 0.2 μM for 4-HNE, 41.8 ± 10.7 μM for FTS and 85.4 ± 19.8 μM for URB597 (n = 4–8). Compared to AITC, the maximal signals were 98% for CA, 82% for 4-HNE, 117% for FTS and 84% for URB597, respectively. Hence, TRPA1 activation by different agonists all induced Yo-Pro uptake.

Several small molecule inhibitors of TRPA1 have been described recently, including AP18, HC-030031 and ruthenium red (RR) [15,16]. We tested whether these antagonists blocked Yo-Pro uptake. AP18 attenuated 30 μM AITC-induced Yo-Pro uptake in a concentration-dependent manner, with an IC_50 _of 10.3 ± 0.8 μM (Fig. 2C and 2D). Likewise, HC-030031 and RR also completely blocked Yo-Pro uptake (IC_50_: 9.4 ± 0.6 μM for HC-030031 and 10.0 ± 1.6 μM for RR). Taken together, these data show that agonist-evoked Yo-Pro uptake is related to TRPA1 channel activities.

**Figure 3:**
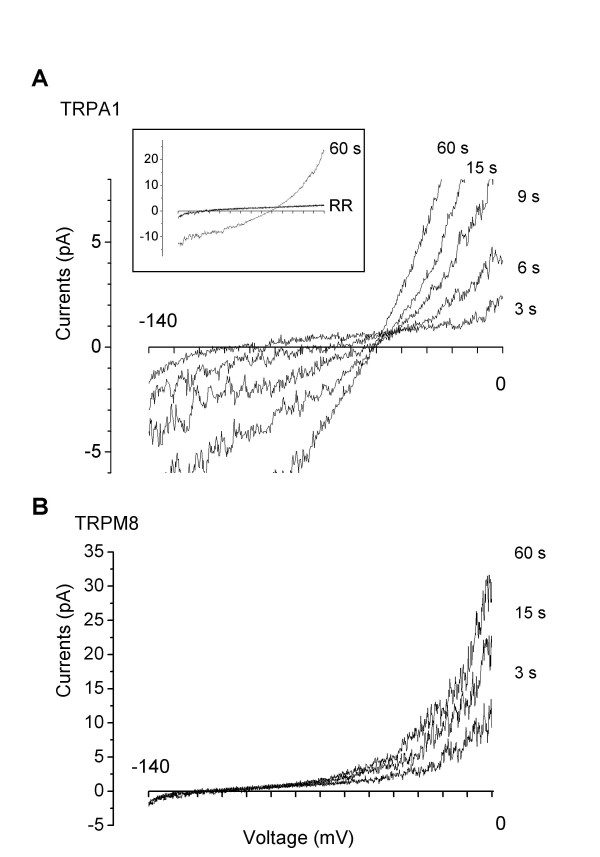
**Ionic currents of TRPA1, but not TRPM8, exhibited shifts in reversal potential**. Outside-out patches were formed from HeLa cells expressing rat TRPA1 or human TRPM8 plus GFP. NMDG^+ ^was the sole external cation and Na^+ ^was the major internal cation. Membrane potential was held at -80 mV, and a voltage ramp from -140 to 0 mV (500 ms duration) was applied every 3 s immediately. A, current traces from a representative TRPA1-containing patch during 60 s application of AITC (100 μM). To illustrate shifts in E_rev_, only currents between -6 to 8 pA were plotted. Inset shows the AITC-evoked currents were almost completely blocked by 10 μM RR. B, currents from a representative TRPM8-containing patch during 60 s application of menthol (500 μM). Note the shifts in E_rev _for TRPA1, but no shift for TRPM8.

Next, we investigated whether TRPA1 undergoes changes in ion selectivity upon channel activation. Currents were recorded under the outside-out patch configuration using NMDG^+ ^as the sole external cation and Na^+ ^as the major internal cation. Patch membrane potential was held at -80 mV, and a ramp voltage from -140 mV to 0 mV (500 ms duration) was applied every 3 seconds. Before addition of AITC, a small basal current was present, consistent with previous reports [17,18]. The reversal potential (E_rev_) of basal currents was -95.3 ± 4.8 mV (n = 5). Compared to activation of TRPV1 by capsaicin, activation of TRPA1 by AITC was relatively slow, probably due to the covalent reaction that is needed to activate TRPA1. Addition of AITC (100 μM) elicited gradual activation of TRPA1 and rightward shift in reversal potential (Fig. 3A). The shift in E_rev _occurred as early as 6 s following addition of AITC, and continued to increase with nearly maximum shift at ~15 s. Addition of 10 μM RR nearly completely blocked AITC-evoked NMDG^+ ^and Na^+ ^currents (Fig. 3A inset), indicating the observed currents were mediated by TRPA1 channels.

**Figure 4:**
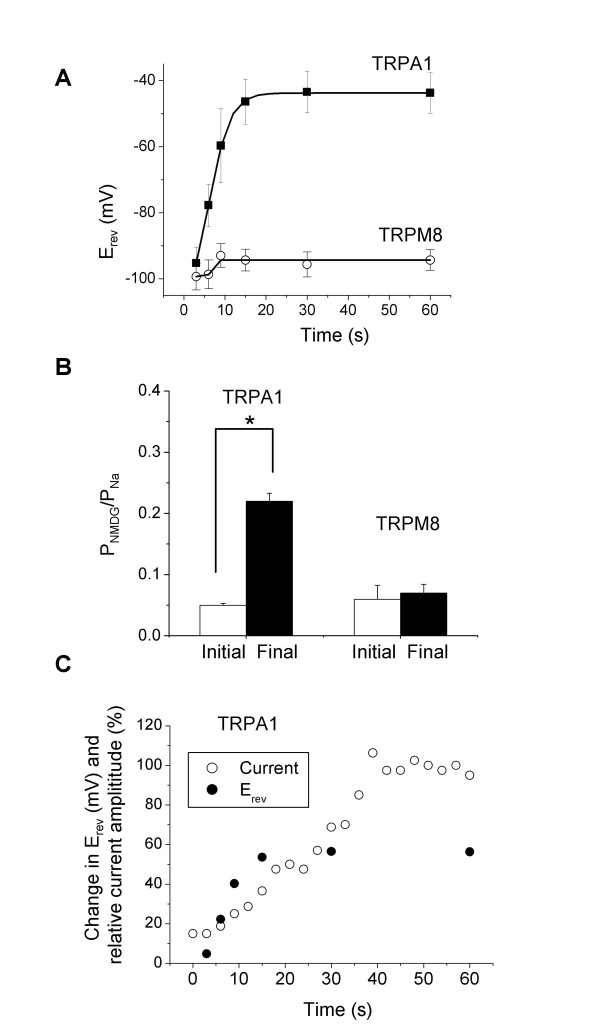
**Time-dependent changes in ion permeability occurred in TRPA1 but not in TRPM8**. A, E_rev _values were determined (from Fig. 3 experiments) and plotted as a function of time after application of AITC or menthol. B, permeability ratios (P_NMDG_/P_Na_) before and 60 s after agonist addition. Student's t-test was used with p < 0.05 as the criterion for significance (indicated by *). C, changes in E_rev _and relative currents at 0 mV (from representative recordings) were plotted as a function of time after application of AITC. Outward currents were measured at 0 mV and normalized against the current obtained after 60 s addition of AITC.

In contrast, TRPM8 showed no shift in E_rev _following addition of 500 μM menthol, despite a clear increase in current (Fig. 3B). The time-dependent changes in E_rev _for TRPA1 and TRPM8 following their activation are shown in Fig. 4A. The shift in E_rev _for TRPA1 was not due to an increase in anion selectivity, as removal of Cl^- ^in the bath solution caused a similar shift in E_rev _from -96 mV to -42 mV. From E_rev _values, permeability ratios (P_NMDG_/P_Na_) before and 60 s after agonist addition were derived. As shown in Fig. 4B, P_NMDG_/P_Na _increased ~4.4-fold for TRPA1 from 0.05 ± 0.003 to 0.22 ± 0.013 (n = 4, P < 0.05, paired t-test), comparable to the ~5.5 fold increase reported for TRPV1 [2]. In contrast, P_NMDG_/P_Na _did not change significantly for TRPM8. It is interesting that the shift in E_rev _occurred much earlier than the increase in TRPA1 currents (Fig. 4C), indicating that pore dilation occurs well before maximal channel activation.

The accuracy of E_rev _measurement could be compromised by small current amplitudes, especially for basal currents and currents immediately following AITC application. However, the E_rev _of basal currents was consistent across patches (-95.3 ± 4.8 mV, n = 5), and E_rev _shifts consistently occurred in TRPA1, but not in TRPM8. In addition, even for relatively large TRPA1 currents, significant shifts in E_rev _occurred. For example, the shift in E_rev _was 31.6 mV between 6 s and 15 s pulses, and 14.2 mV between 9 s and 15 s pulses (Fig. 3A and 4A). Taken together, these data suggests that the dynamic change in E_rev _results from TRPA1 channel activity. Another concern in extrapolating the change in E_rev _to the change in ion selectivity is that ion accumulation can occur during prolonged activation, particularly when large currents are conducted under whole cell configuration. However, the ion accumulation should not significantly compromise our TRPA1 experiments using outside-out patch configuration, in which extracellular and intracellular ionic conditions were well controlled. In addition, reversal potentials changed within seconds of AITC application when currents were small, but reached a steady state (from 15 to 60 s) when currents were relatively large (Fig. 3A and 4A). Furthermore, TRPM8 conducted currents with similar amplitudes but without significant shifts in reversal potential. Consistent with the electrophysiology data, the large divalent cation Yo-Pro did not cross the membrane when the channel was closed or blocked by antagonists, but permeated the membrane freely when the channel was open (Fig. 1B and 2C). Collectively, our data suggest that TRPA1, but not TRPM8, undergoes pore dilation.

Pore dilation has been previously described for the ATP-gated P2X and TRPV1 [2,8,9]. For P2X channels, the mechanism underlying pore dilation remains controversial. Several alternative mechanisms have been proposed including a direct change in ion selectivity, formation of channel multimers, and recruitment of a downstream, nonselective pore [19,20]. For TRPV1, pore dilation most likely arises from a change in ion selectivity, as indicated by the dynamic change in ion selectivity during agonist stimulation, and the effects of mutations and chemical modification of certain residues within the selectivity filter [2]. In the current study, we did not elucidate the biophysical mechanism underlying pore dilation of TRPA1. However, there were several notable observations. First, the permeability to NMDG^+ ^increased almost immediately upon channel activation. Second, the outside-out patch configuration should largely disrupt cytoskeletal structures and washout cytosolic factors. Third, the AITC evoked- NMDG^+ ^and Na^+ ^conductance was sensitive to blockade by ruthenium red. Finally, under identical conditions, TRPM8 conducted large currents, but did not exhibit Yo-Pro uptake or a significant change in NMDG^+ ^permeability. Thus, TRPA1 pore dilation most likely represents a direct change in ion selectivity. Nonetheless, our present study does not completely rule out the involvement of other proteins.

TRPV1, TRPA1 and TRPM8 are major TRP channels involved in somatosensation. Within dorsal root ganglia, TRPV1 and TRPA1 are co-expressed and interact functionally in one population of sensory neurons, while TRPM8 is expressed largely in a separate neuronal population. Interestingly, pore dilation occurs in TRPA1, TRPV1 but not TRPM8, suggesting that this property is not ubiquitous, but rather specific to subtypes of channels within a subpopulation of neurons. The change in cation permeability, in turn, may alter channel function, affect a host of downstream processes (e.g., neurotransmitter release, cellular toxicity) and contribute to pain hypersensitivity [21]. Recently, it was reported that TRPV1-mediated pore dilation could be utilized to deliver QX-314 (a membrane-impermeant sodium channel blocker) specifically to TRPV1-positive sensory neurons, achieving analgesic effects without motor deficits associated with local anesthetics [5]. However, this strategy of targeting TRPV1-positive neurons could be compromised by several factors, including the broad expression pattern of TRPV1, its role in regulating body temperature, and its involvement in hippocampal synaptic plasticity [22,23]. By analogy to TRPV1, the pore dilation of TRPA1 could be exploited to mediate entry of QX-314 specifically into TRPA1-positive neurons. Given the restrictive expression of TRPA1 in sensory neurons, this strategy may offer analgesic efficacy without unwanted side effects.

In conclusion, the present study demonstrates that pore dilution occurs in TRPA1 but not in TRPM8 channels. This finding raises many interesting questions: What is the exact biophysical mechanism underlying pore dilation of TRPA1? What are the physiological, pathological and therapeutic implications? Why does pore dilation not occur in TRPM8? What are the pore behaviors of other TRP channels? Answers to these questions will certainly extend our understanding of this family of ion channels.

## Methods

### Transient expression of recombinant TRPs

Full length cDNAs for rat TRPA1(GenBank Accession: NM_207608), human TRPA1 (NM_007332), mouse TRPA1 (NM_177781) and human TRPM8 (NM_024080) were cloned into pcDNA3.1/V5-His TOPO vector and transiently expressed in HEK293-F or HeLa cells [24]. For the Ca^2+ ^influx or Yo-Pro uptake assay, HEK293-F cells were transfected with TRP cDNA, collected 48 hours post transfection, and used either fresh or following storage at -70°C. For electrophysiological experiments, HeLa cells were transfected with TRPA1 or TRPM8 plus GFP, and used 48 hours later.

### Ca^2+ ^influx and Yo-Pro uptake assays

Ca^2+ ^influx assay was performed using the FLIPR™ and calcium assay kit R8033 (MDS Analytical Technology) as reported previously [25]. After incubation with 100 μl of 1 × Ca^2+ ^dye for ~2 hours at room temperature, a two-addition protocol was used for evaluating agonist activities (i.e., activation of Ca^2+ ^influx) and antagonist activities (i.e., inhibition of agonist responses): 10 s baseline readout, addition of 50 μl assay buffer or antagonist (4 × stock), 3–4 min readout, addition of 50 μl agonist (4 × stock), and readout for 2.5 min. Maximum minus minimum signals before the second addition and at the end of the experiment were obtained.

Yo-Pro uptake was determined using the FLIPR™ and Mg^2+^/Ca^2+^-free DPBS buffer as reported previously [26]. Briefly, immediately after loading with 100 μl Yo-Pro dye (2 μM), a two-addition protocol was used for evaluating agonist activity (i.e., Yo-Pro uptake) and antagonist activities (i.e., inhibition of agonist evoked Yo-Pro uptake): 10 s baseline readout, addition of 50 μl assay buffer or antagonist, 3 min readout, addition of 50 μl agonists, and readout for 60 min. Max-min fluorescence signals before the second addition and at the end of the experiment were obtained.

### Outside-out patch recording

Before forming cell-attached patches from HeLa cells expressing TRPA1 or TRPM8, pipette offset was adjusted to give a zero current value. Outside-out patches with the least amount of leak were used, as judged by the very small DC shift (<5 mV) of the basal current at different membrane potentials. Currents were recorded using AxoPatch200B. The pipette solution contained (mM): 140 NaCl, 1 MgCl_2_, 5 mM EGTA, and 10 HEPES (pH 7.3). For experiments with NMDG^+^, the bath solution contained (mM): 150 mM NMDG^+^, 115 mM Cl^-^, 5 mM EGTA, and 10 HEPES (pH 7.3). For Cl^- ^replacement experiments, the bath contained 150 mM NMDG^+^, 62 mM EGTA and 10 HEPES (pH 7.3). Patch membrane potential was held at -80 mV, and then a voltage ramp from -140 mV to 0 mV (500 ms duration) was applied every 3 seconds. Current was filtered at 1 kHz using 8-pole Bessel filter (-3 dB; Frequency Devices) and transferred directly to a computer using the Digidata 1320 interface (Axon Instruments) at a sampling rate of 10 kHz. Permeability ratio (P_X_/P_Na_) was calculated using the equation: P_X_/P_Na _= ([X]_o_/[Na]_o_)• exp(ΔE_rev _•F/RT); where ΔE_rev _represents the shift in E_rev _after addition of AITC in NMDG^+ ^external/Na^+ ^internal solution, and F/RT is 0.040 mV^-1^. The activity coefficient of Na^+ ^and NMDG^+ ^was taken as 0.75 and 0.81, respectively. Student's t-test was used with p < 0.05 as the criterion for significance. Data are represented as mean ± S.E. unless specified otherwise.

## Competing interests

The authors declare that they have no competing interests.

## Authors' contributions

JC conceived, coordinated the study and made the initial finding. BRB and JC conducted FLIPR experiments. DK and EJC conducted the electrophysiology experiments. CRF, PRK and RMR supported the study. JC and DK wrote the manuscript. All authors read and approved the final manuscript.
